# The Use of Drones in Spain: Towards a Platform for Controlling UAVs in Urban Environments

**DOI:** 10.3390/s18051416

**Published:** 2018-05-03

**Authors:** Pablo Chamoso, Alfonso González-Briones, Alberto Rivas, Federico Bueno De Mata, Juan Manuel Corchado

**Affiliations:** 1 BISITE Digital Innovation Hub, University of Salamanca, Edificio Multiusos I+D+i, Calle Espejo 2, 37007 Salamanca, Spain; alfonsogb@usal.es (A.G.-B.); corchado@usal.es (J.M.C.); 2 Faculty of Law, University of Salamanca, C. Miguel de Unamuno. P. Francisco Tomás y Valiente, s/n. 37007 Salamanca, Spain; febuma@usal.es; 3 Department of Electronics, Information and Communication, Faculty of Engineering, Osaka Institute of Technology, Osaka 535-8585, Japan; 4 Pusat Komputeran dan Informatik, Universiti Malaysia Kelantan, Karung Berkunci 36, Pengkaan Chepa, Kota Bharu 16100, Kelantan, Malaysia

**Keywords:** Unmanned Aerial Vehicles, secure uav platform, law and policy, Ground Control Station

## Abstract

Rapid advances in technology make it necessary to prepare our society in every aspect. Some of the most significant technological developments of the last decade are the UAVs (Unnamed Aerial Vehicles) or drones. UAVs provide a wide range of new possibilities and have become a tool that we now use on a daily basis. However, if their use is not controlled, it could entail several risks, which make it necessary to legislate and monitor UAV flights to ensure, inter alia, the security and privacy of all citizens. As a result of this problem, several laws have been passed which seek to regulate their use; however, no proposals have been made with regards to the control of airspace from a technological point of view. This is exactly what we propose in this article: a platform with different modes designed to control UAVs and monitor their status. The features of the proposed platform provide multiple advantages that make the use of UAVs more secure, such as prohibiting UAVs’ access to restricted areas or avoiding collisions between vehicles. The platform has been successfully tested in Salamanca, Spain.

## 1. Introduction

Unmanned Aerial Vehicles (UAVs) are not new; they have been in existence for dozens of years, however only recently have they become popular. With the development of technologies, modern UAVs have also become more advanced and our possibilities for using them have become greater, which poses new legal and regulatory questions. Initially, they were designed for military purposes: to transport balloon bombs and as a form of training for anti-aircraft weapons during World War II. Today, their use is becoming more frequent and they can carry out a wider range of tasks in both the military and professional sectors. This second case is the focus of this article. The popularity of using UAVs for professional tasks was largely achieved thanks to the appearance of multirotor systems about eight years ago, a new type of UAV that offers great benefits and advantages over the UAV and manned systems used up to that time.

Some of the advantages of these systems over other types of UAVs are their mechanical simplicity, greater stability and tuning. In comparison to UAV aircraft, rotorcraft do not need a runway to take off since they can lift vertically off the ground. Traditional UAV helicopters are not as stable as multirotors are and they require complex mechanical components to achieve stability. On the other hand, stability can be achieved much more simply and effectively in multirotors, which use speed variations in their motors for this purpose.

UAV systems have many advantages over other manned aerial systems, especially when used in the professional or commercial fields. Their cost is lower; they are portable (smaller in size); and they can fly in small, enclosed or crowded areas. For example, they can fly in urban areas at low altitude or even inside buildings. Moreover, the fact that UAVs are steered remotely is also a big advantage; no persons on board means that, in the case of it crashing, no people would be harmed.

The chance of a UAV falling is minimal, but possible and can be caused by several factors, such as collision with an object or some error of the pilot. However, there is also the possibility that pilots purposefully use a UAV to cause damage or infringe the privacy of citizens.

The problem with flying devices is that they can easily access private and restricted environments. There are no legal, nor physical barriers that would prohibit their access, which means that UAVs can be used to intrude peoples’ privacy and there is uncertainty about whether this practice is legal.

Different proposals have been made that attempted to regulate the use of UAVs and their access to certain areas by proposing measures such as licensing or banning urban hubs, however these measures often limit the possibilities that technology offers. Moreover, legislation varies from region to region and between countries.

This article takes a proactive approach towards state of the law in Europe, especially in Spain. In this paper, we present a platform that has been developed under this legal framework. UAVs can connect to this platform, which monitors them to comply with all the laws. For example, competent authorities can monitor and control UAVs through the platform in real time. In this way, suspicious behavior can be detected, access to restricted areas can be controlled, security protocols can be added to prevent the pilot from losing control or signal, autonomous flights can be created or collisions between the UAVs connected to the platform can be avoided.

The rest of the article is structured as follows: Section 2 provides a brief review of the state of the art on UAVs. Section 3 analyzes current European law, focusing on the legislations in Spain in more detail. Section 4 describes the proposed system. Section 5 shows the results obtained when the proposed system was tested with UAVs. The last section presents the conclusions drawn from the conducted research work and possible lines of work for the future.

## 2. Brief Background

Over the last few years, the use of UAVs in surveillance and civilian tasks has increased significantly [1]. Moreover, UAVs will have more applications in the future. The rapid advances in technology allow reducing the size of devices and their cost. All of this makes it possible to use UAVs in ways that would have been unimaginable 10 years ago.

Among their many applications, UAVs can be used for social purposes such as search and rescue operations [2], automatic forest fire monitoring and measurement [3] or disaster management [4]. Commercial tasks also appear within sectors such as agriculture [5], construction [6], mapping and geology [7], surveillance [8] or in the cinema industry [9].

In comparison to other alternatives, the low cost of new UAVs and the wide range of areas to which they can be applied have cause great interest in this technology. They have become so widespread that it is necessary of legislate the use of UAVs to prevent illegal acts and accidents.

Some studies in the literature analyzed the legal aspects of using robots and autonomous systems in fields such as video surveillance, image rights and defence. These complex issues are already being put on the agenda to manage the deployment and operation of UAVs as their technical expertise evolves. One of the first serious analysis of these legal and ethical issues was conducted by Finn and Scheding [10].

However, an analysis of the wide range of functionalities provided by UAVs is not sufficient for developing a platform that would monitor UAV flights. It is necessary to consider the legal aspect when developing the platform, since all the rules that regulate the use of these vehicles must be encompassed by the developed platform.

## 3. Current Legislation in Europe Regarding UAVs and Its Legal Transposition into Spanish Law

This section describes the legislation in Europe and Spain, especially the Spanish laws that were passed in December 2017.

The European Union has to create new laws as information technologies evolve. The continuous progress in technology is a challenge to EU policies and regulations. This is because technology provides us with increasing possibilities that must be regulated under the law to protect citizens’ rights and interests.

Several institutions have already been set up in Europe to work on these issues, however our project goes much further. In this context, the European UAV market is growing larger every year. To date, at least sixteen of the twenty-eight EU Member States already use UAVs for military and police purposes. The EU placed security projects as one of the priorities on its agenda and began to invest in UAV proposals.

In 2012, a report on Aeronautic Industries announced a plan for developing UAV legislation for both commercial and safety purposes. This report pointed to the emerging UAV market and expressed the need for regulating the operation of these systems.

The next step was taken in 2017 when the European Commission published a report on UAVs [11]. In it, it set the alignment of different national UAV legislations as one of Horizon 2020 goals and it called on the individual member states to adopt measures that will allow for the integration of these systems in the European civil airspace. However, this goal has been postponed until 2050 [12].

Making progress on the issue of reaching to a common legislation will be a complicated task. This is because international conventions on international civil aviation, such as the Chicago Convention, apply only to civil aircraft but not to State aircraft, the latter defined as devices used in military, customs and police services, including UAVs used by public administrations for the protection and restoration of the environment. However, article 8 of the Convention does not allow any unmanned aircraft to fly over the territory of any State without the authorization of that State.

As a result of article 8 of the Convention, the priority of aviation institutions such as ICAO (International Civil Aviation Organization), EUROCONTROL (European Organization for the Safety of Air Navigation) or EASA (European Aviation Safety Agency) must be to promote European regulation. This would be key for updating conventions in search of a comprehensive directive.

Following this first indication, European institutions should promote aviation regulation; in 2014, the Commission published “A new era for aviation—Opening the aviation market to the civil use of remotely piloted aircraft systems in a safe and sustainable manner” [11]. According to this document, by 2016, the EU should have adopted measures to allow for the integration of these systems into civil airspace.

However, security responsibilities are left in large part to the Member States. That is why UAVs used for police purposes are still excluded from any current European legislation, since it has resulted in very disparate regulations (if any) in the different EU states with regard to these systems and harmonization in this respect is necessary. It is necessary to have a legislation that will be open and generic in the technical aspects. This is because legislations that are limited to specific aircraft types or only permit the use of remote controls with certain characteristics would become obsolete in the near future, as new advances in the field of UAVs appear. In addition, they could easily lead to confusion and legal problems. Furthermore, regulations should not only consider the civil liability of these devices but also aspects that will assure the security of the state and of citizens, for example the protection of data in deployed vehicles. These regulations should also indicate the legal consequences of inadequate use or combining their military and police uses. Similarly, proceduralists should be called to clarify all issues, such as the use of UAVs as a source of evidence or their use within the limits set by the jurisdiction of each state: solving this issue is a priority when environmental crimes, such as arson, occur on transboundary lands.

However, current legislations do not deal with the use of aircraft in the correct order. Law 18/2014, or Royal Decree 601/2016, of 2 December, which approves the Regulation on Operational Air Traffic, dedicating its Ninth Book to unmanned aerial systems [13]. As Marquez Lobillo described about Law 18/2014, “it is far from establishing a complete legal regime that would facilitate the integration of these aircraft, the rules it provides for rules are partial and incomplete” [12]. This law has been updated recently, making Spain a pioneering legal system in UAV legislation. Since 15 November 2017, the new Royal Decree allows the use of UAVs at night and their flight over urban areas, which had until then been prohibited. Spain now has a much more inclusive and flexible regulation that at the same time seeks to preserve the rights and privacy of every citizen.

The Royal Decree 1036/2017 of 15 December 2017 came into force in 2018 upon the publication of the “Strategic Plan for Drones”, a regulatory framework drawn up by the Spanish Ministry of Public Works. This new legislation regulates the civil use of remote-controlled aircraft by private individuals for recreational and leisure purposes, while also incorporating provisions for the use of these devices by the Judicial Police.

These changes have led us to a new procedural status: the purpose of using drones is to collect audiovisual evidence which may only be acquired by respecting the fundamental rights of those under investigation. Moreover, for such evidence to be valid, it must fulfill a series of characteristics, without infringing procedural guarantees and principles.

Despite this beneficial legislation, we believe the regulations of all countries should encompass not only civil, but also criminal and procedural aspects, which would establish regulations for the use of drones in police investigations or by different branches of government. This would be important as by 2019 Brussels is planning to create “Uspace” [14], a European controlled space for drones flying above 150 m in height and weighing less than 150 kg. It seems that future European legislation will already address fully automated models, i.e., models that operate without a remote control and are equipped with safety measures to avoid obstacles and collisions, in the same way as intelligent cars. These last-mentioned issues are still outside our regulations, since, as we indicated, it is only designed for remotely piloted aircraft and where automation by means of artificial intelligence does not apply. Initially, this space will not affect the drones regulated under this legislation, since it will not be designed for drones that incorporate artificial intelligence technology and that will have sensors to detect obstacles and thus perform a free flight [15].

Thus, a complete regulation would specify the use of RPAS by different subjects. Professionals and legal operators would be able to use these devices as technological research diligences, and civilians would only use them for leisure or recreational purposes. However, in all cases, these would be civilian drones, capable of taking images and filming. They incorporate specialized technology such as thermographic scanners. All these issues make us think of the legal implications of drones.

### The UAV as Evidence in Court Reflections on Its Incorporation into the Legal Process According to Spanish Law

UAVs can also be used in crime investigations, for making photographic or video documentations of the crime scenes. They are effective tools that allow performing a proper examination of the scene and obtain evidence quickly. Such electronic evidence can help determine the circumstances of the crime or identify the perpetrator. However, the use of UAVs for legal purposes has raised many doubts since the legislator often fails to decouple the concept of electronic evidence (“Any material support that expresses or incorporates data, facts or narratives with evidentiary effectiveness or any other type of legal relevance” —Article 26 of the Spanish Penal Code), since we often dissociate the physical element of the UAV from the intangible information that lies in it, whereas we should really refer to a common concept based on a binomial between a physical and a logical part.

From this point of view, we would deal with two sources of evidence: the device and the information contained within it. The UAV as a whole should be considered as electronic evidence, thus we must refer to obtaining the images captured with the UAV in such a way that they do not infringe the fundamental rights of the person under investigation.

In this regard, the enactment of a regulation on the collection of electronic evidence on the European grounds is urgent, as it allows for subsequent transposition into national law, underpinning this decree on the ISO/IEC 27037:2012 standard “Information technology—Security techniques—Guidelines for identification, collection, acquisition and preservation of digital evidence” [16], which establishes minimum standards that could easily be implemented.

Having dealt with the issues of obtaining and securing evidentiary material, we now have to consider the types of evidence that a UAV can provide in a judicial process. UAVs can make photographic or video documentations which could be recorded words, sounds or images, as regulated under art. 299.2 of the Spanish Civil Procedure Law (Ley de Enjuciamiento Civil Española), which states that “according to this law, recorded images, words and sounds are admitted as evidence, as well as the devices which allow to archive, recognize or reproduce words, data, figures and mathematical operations carried out for accounting or other purposes and which are relevant to the process”. There is also a supplement to this law, called art. 382 LECiv., which further regulates the practice. It states that “The parties can provide as source of proof, before the court, words, images and sounds recorded with a filming or recording device or other similar” and “The party providing such evidence to the court, should also hand a written transcript of the words contained in the recorded evidence, whenever this applies and which are relevant to the case”.

On the other hand, if we want to include the device itself as evidence, we should refer to art. 384 LECiv, which states that “the devices that allow to archive, recognize or reproduce words, data, figures and mathematical operations carried out for accounting or other purposes, which are relevant to the case, will be admitted as evidence and shall be examined by the court with tools provided by the proposing party or which the court may decide to use and in such a way that the other parties involved in the case may, with the same knowledge as the court, assert and express what is in their best interest”. Moreover, the second point states that “the documentation should be stored on file in a way that is most suited and where the court clerk shall also, where appropriate, adopt custody measures”. In this regard, not only an examination of the “content” is necessary but also of the “device” storing this evidence. This will allow verifying that the submitted evidence is accurate and authentic. Moreover, the other party can submit and propose whatever it deems appropriate in this respect, in accordance with the principle of contradiction.

In this way, external part of the UAV becomes part of the evidence presented in a court process, as a device used for archiving data. The UAV must be presented to the judge directly, that is, through judicial recognition of the hardware, which in turn is supervised by the Attorney of the Administration of Justice. Likewise, when referring to a penal level, the articles of the Criminal Code must be applied, i.e. 334 and 338 LECRim, in the sense that it is necessary to guarantee the integrity and retention or conservation of impounded materials that can serve as evidence in a trial and thus preserve the device from any subsequent manipulation through the application of any securing technique. Finally, in both cases, an examination of such device is by means of an expert opinion, whenever the knowledge of an expert is required, to give a purely technical opinion on the software and hardware of the unmanned aerial vehicle as well as the LIDAR technology mentioned above.

In conclusion, irrespective of the source of evidence, a prior admissibility check on the grounds of the lawfulness, legality, relevance and usefulness of evidence is required. Subsequent practice would have to respect the relevant procedural guarantees and principles.

## 4. Proposed System

At the technical level, therefore, we will still have to wait for some time until commercial UAVs can be adapted to a judicial process, such as the one considered in the previous section. However, our work with UAVs started back in 2011 and much of our time has been dedicated to the development of a software platform that would monitor and record the parameters obtained by connected UAVs in a central system.

The main objective of this work is to make the developed platform suitable for type of existing commercial UAV, so that they can enjoy added functionality (autonomous flights, different security mechanisms, telemetry storage, etc.), while ensuring that all their flights abide to current law. Thus, when developing this system, we first analyzed the different existing UAV systems and looked for a way to integrate them in a single platform. The best solution is to design a module capable of communicating with our platform, integrating all the functionalities, and enabling the control existing types of commercial UAV as if the platform itself were the pilot. The chosen solution is described in more detail throughout this section.

To connect with the platform, only one hardware module must be incorporated into the UAV. This module connects with the communication infrastructure deployed in the city and allows the UAV to be controlled in a traditional way, with radio controllers or commercial Ground Control Stations (GCS). UAVs can also be controlled manually or autonomously through the developed software. A gamepad is used for manual control, while configurable waypoints or advanced behaviors are defined for autonomous control.

Wi-Fi communication is the solution for this platform, it allows to monitor all telemetry of the connected aerial vehicles and control them, when their behavior has to be altered for security or privacy reasons. The use of Wi-Fi communication is novel here since the telecommunication systems traditionally used for the control of these vehicles are long-range analog radio communication systems. These systems are useful for the transfer of long-range information between two points (which in this case are the pilot and the aircraft), but present the problem in the poor video quality and the limited variety of file formats allowed for the information they transmit.

On the other hand, Wi-Fi connectivity makes it possible to establish totally secure connections, not only point-to-point, but also multiple devices and users can be connected. In addition, they provide sufficient bandwidth for two-way transmission of digital information, allowing even high-definition, real-time video to be transferred. The only drawback of using Wi-Fi-based systems is that their range is considerably more limited than the long-range systems mentioned above.

However, this problem can be solved by establishing different access points as repeaters of the Wi-Fi signal. This would allow the vehicle to connect to the point with the strongest signal at all times. In this way, simple studies can be carried out to guarantee connectivity throughout the entire authorized flight territory. Although the signal of Wi-Fi antennas can nowadays reach long distances, the current Spanish legislation does not allow pilots to separate themselves from the UAVs.

To connect any UAV to the platform, it is only necessary to install the developed module. This allows connecting to the system’s Wi-Fi network and exchange all kinds of data: flight commands, telemetry, route points and associated behavior and HD video.

**Figure 1:**
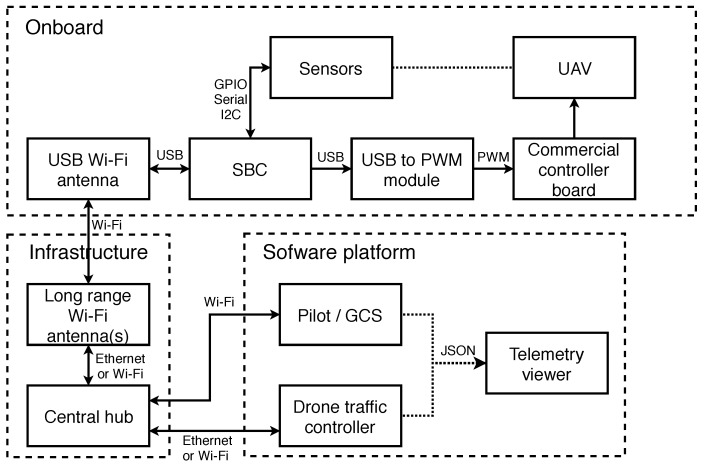
Global system connection diagram for a UAV.

This module is an SBC (Small Board Computer), more specifically a low cost and lightweight Raspberry Pi, based on the Linux operating system connected to a powerful Wi-Fi antenna. This module is in charge of translating the information received from the pilot, the person who is in charge of the security of the system or the platform in the event of automatic flights or changes in behavior due to security (arrest in the case of wanting to access a restricted area). The information is converted to PWM (Pulse Width Modulation) signals, which is the type of information that commercial UAVs understand and allow their control (movements, flight modes, etc.). A schematic diagram of the platform connection for a single UAV can be seen in Figure 1.

Any number of sensors can be connected to the SBC, which can later be monitored from the platform or from the pilot’s control station. The pilot can optionally choose to control the flight using the developed software, which allows for manual control using a gamepad that is much simpler than complex radio stations. The software also makes it possible to program an autonomous flight by previously defining the behavior of the UAV.

**Figure 2:**
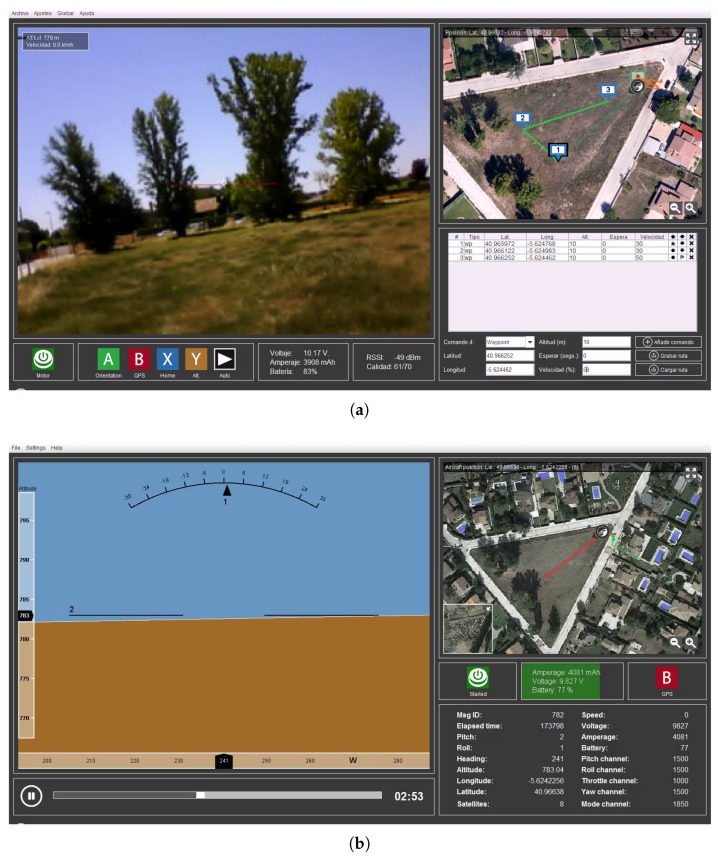
Developed software: (**a**) ground control station; and (**b**) recorded telemetry system.

In addition to the software running on the control base station, as shown in Figure 2a, we have developed the software that the UAV traffic controller uses. In this software, the UAV traffic controller can select which UAV they want to monitor or control individually; they can also use different pre-defined behavioral options, such as stop, landing at source, landing at a defined safety point, and so on. Users can also set the viewing order to be able to see all the connected UAVs on the map, with graphical aids which help to easily determine their height, for example.

The system ensures that the aircraft does not access unauthorized locations as this would be an infringement of the law. It also prevents the aircraft from flying to areas with poor connectivity can pose problems of scope. This is done by analyzing the position of the aircraft each time a message is received, and by checking the bounds of the non-permitted zones. When an aircraft approaches these boundaries (between 0 and 20 m), a warning with an icon is displayed both on the pilot’s screen and on the controller’s screen. If the pilot intends to enter an unauthorized, their movements are ignored. Similarly, in the event that any external force (other than the pilot’s orders) moves the aircraft into an impermissible area, the aircraft will be moved straight ahead and automatically from the controller software to the point on the edge closest to its position.

All the information transmitted can be recorded and stored by both, the control station used by the pilot and on the control platform (in this latter case it is mandatory). This information is recorded securely and encrypted, in JSON format, and allows viewing all telemetry received from the developed third-party software, in this case only to “play” the JSON file as if it were a video, where all the information is presented on an interface that is very similar to that of the flight in real time. Figure 2b shows a screenshot showing the status of all parameters, at a certain time of a past flight.

Each message exchanged during the flight of a UAV is encrypted with blockchain technology so that a new message depends on each and every one of the messages sent previously. This is achieved by adding a field in the transmitted message (*n*) that consists of a hash obtained from the encryption of the previous message (n−1). The previous message in turn, will contain in that field the hash obtained from the encryption of the message n−2 and so on until reaching the very first message, which is encrypted from the timestamp of the cloud environment in which the air traffic controller software is deployed.

Due to the use of this technique, the system obtains a valid telemetry document. Despite being stored in a plain text it cannot be modified. If one of the values is changed in the *m* message, the entire sequence of messages obtained from that message (from m+1 onwards) will have a visibly incorrect value in the field containing the hash.

The transmission mode is based on ACK. If a message is not delivered, the ACK signal is not received from the controller (the pilot does not send ACK messages), so that information is sent again until the ACK message is received. Only the message information with the highest timestamp is displayed, so the outdated information will not be displayed. If a message is received in a disorderly manner, it is inserted into a message queue in an orderly manner in the corresponding position. Messages with flight orders follow a similar pattern. However, in the case the pilot indicates a flight mode change, a A SYN-ACK model is followed in the message with an order.

Thus, the platform does not only provide security but can also be used in legal issues, which is a very important aspect. The messages can be used in a judicial process since the use of blockchain assures that information has not been altered and if such attempt has been made it can be easily detected.

The messages exchanged between the UAV and the platform (both with the controller software and with the pilot software) follow a similar scheme to that used by the well-known MAVLink protocol (secured version [17]), although the protocol designed for communication is smaller in terms of the type of messages that can be exchanged, since their payload can be configured according to the parameters of the header, with the freedom to send structured information with a single type. More specifically, there are message types for transmitting flight orders, flight modes, sensor information (adaptive type and payload), UAV position and orientation, pilot identification, UAV identification, flight configuration, UAV configuration and UAV type.

**Figure 3:**
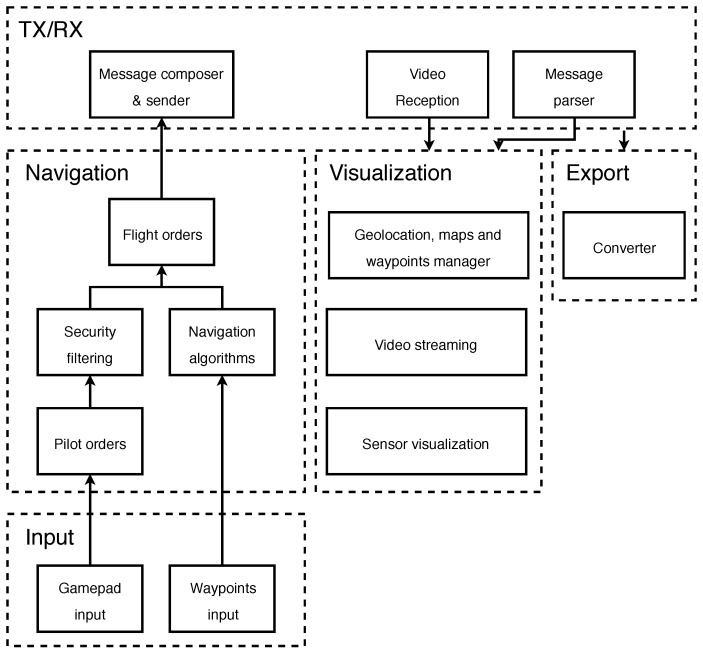
Developed software modules.

All the software has been developed in Java, so it can be run on different platforms. It has been successfully tested on MacOS, Windows and Ubuntu. Although there are three independent applications (pilot, controller and telemetry display), there are layers common to all of them that are integrated following the scheme presented in Figure 3.

In the “TX/RX” layer, the functionality of processing the received information is implemented, thus it is integrated in the three designed applications. However, the real-time information is processed by the pilot and controller software. The “Navigation” layer, which is also integrated in the pilot’s software and the controller’s software, including all the algorithms for translating the pilot’s movements into flight orders, as well as algorithms for translating autonomous flight movements into flight orders. The functionality of the “Input” layer allows the pilot to directly indicate the movements they want to make, through a gamepad or by creating waypoints on a map. Therefore, this layer is only integrated into the software used by the pilot to control the ship. The “Visualization” layer is incorporated into all three software applications. It presents all the information that is to be displayed in the UAV. The “Export” layer is responsible for generating the corresponding telemetry files so that they can be processed by the telemetry visualization software after each flight.

## 5. Experiment and Results

Having designed and developed the system system, it was necessary to test all its functionalities in a real environment, in addition to evaluating other aspects such as the scope, response time and the performance of different types of commercial UAVs. This section details, the experiments performed for validation.

The proposed system was tested and evaluated in the city of Salamanca, Spain. It is a small town of 39.34 km${}^{2}$. Infrastructure was deployed in this city, consisting of 36 points with 4 Wi-Fi antennas at each point. The antennas used were Ubiquiti NanoStation M2 antennas, which have a unit price of 80€. Different existing tools were used to determine the coverage areas and the best positions for placing the antennas. With the chosen model, an average range of 10 km for each antenna placed with direct view was achieved (obviously, parameters can affect connectivity). Instead of a WiFi antenna, other models can be used as access points, even if they come from another manufacturer. Deployment in a city with these characteristics has not been evaluated on another model as it requires a high initial investment and could be part of future work.

Once the infrastructure was deployed, the area in which the different tests were to be carried out was defined. In this regard, all take-offs had to be carried out in a secure area and the UAVs had to reach a safe height, which for the city of Salamanca was established at 900 m above sea level, approximately 100 m away from the pilot or the control station that operates the UAV. To monitor air traffic, UAV traffic controller software was constantly running in a cloud environment that allows storing all UAV connections to the platform as well as all telemetry was generated during that connection.

The tests were carried out with 8 UAVs of 3 different commercial models, at randomly selected points, which were distributed throughout the city. The time it takes the system to display information in the air traffic controller software is negligible (average of 185 ms relative to the time of representation in the pilot control software). Similarly, the time it took to modify the behavior of the UAVs was also negligible and, therefore, could not affect safety, for example when avoiding collisions between them. In this case, the average time that passed between the flight order being sent and the UAV executing it was 211 ms. Therefore, the reaction time, from the moment a behavior occurs that must be independently rectified by the platform until the UAV actually performs it, is approximately 396 ms.

Therefore, even though the system is distributed throughout the city, the average reaction time that the platform invests in solving incidents autonomously can be said to be much shorter than the average reaction time that pilots would manually invest.

With respect to the number of UAVs that can be connected to the platform without affecting the performance of the overall system, the maximum has not yet been determined, although it is a high number of UAVs given the low bandwidth to be dedicated to telemetry sending: 40 bytes on average per message and 6 messages exchanged per second. That is, each UAV consumes 240 B/s (not counting the sending of the video, optional) and the TX/RX rate in Nanostation M2 is 130/130 Mbps. Thus, we could use thousands of UAVs theoretically.

## 6. Conclusions and Future Work

The developed system allows controlling and monitoring flights and recreating all the past flights found in that system. The system can be easily adapted to the size of the city (the only requirement is to deploy the infrastructure) and it can operate thousands of UAVs simultaneously.

Due to the diversity of UAVs and commercial controllers, the platform was designed to adapt to any type and manufacturer. However, UAVs may vary depending on their manufacturer so it will be necessary to adapt their peculiarities to the platform (it has already been done with the best-selling models and brands, such as DJI). It will remain functional even if the structural and technological side of UAVs changes. It is prepared to withstand technological changes, so more advanced models from new manufacturers can also be integrated with the system through the hardware and software modules, even if the vehicle is not a multirotor, traditional helicopter or airplane.

The use of this system guarantees that the privacy of citizens is protected. This is because the platform defines restricted zones, where UAVs cannot fly; even if the pilot explicitly intends to pass these limits, the platform will prevent them from doing so. The platform uses for this purpose different collaborative and point-based systems. On the one hand, in the collaborative system, anyone can justifiably propose an environment as private. The competent security and privacy authorities shall determine whether to grant such status to the requested area. On the other hand, the points-based system allows giving warnings and penalizing pilots who attempt misconduct.

Therefore, to use the platform, in addition to coupling the developed module, it is essential to be registered as a pilot through an account that is created when obtaining a license in European countries, such as Spain. From the legal point of view, the platform offers a robust mechanism for demonstrating the status of UAVs. This is because blockchain is used in telemetry, making it possible to prove judicially that the pilot controlling the UAV infringed the law. Thus, in addition to offering enforcement mechanisms, the platform also provides mechanisms to demonstrate non-compliance with the law in other cases where non-compliance cannot be independently avoided. Finally, by using this software, the cause of malfunction in a UAV can be determined even if the incident involved a human error or was provoked intentionally.

In the future, we must work on testing what number of UAVs can the proposed system operate simultaneously, by both number of antennas deployed and the number of drones that the overall system is able to control. In addition, we will work for the standardization of the system that will result in the serial production of the module that UAVs must carry to connect to the platform. In series production, the hardware device is expected to reach a price of 35€. We are constantly adapting new models which appear on the market and are popular among UAV pilots, and we intend to continue doing this with model that will be launched soon.
